# Supplementary material for: The nuclear transport factor CSE1 drives macronuclear volume increase and macronuclear node coalescence in Stentor coeruleus
Source: iScience. 2023 Jul 10;26(8):107318. doi: 10.1016/j.isci.2023.107318 (PMC10374459; doi:10.1016/j.isci.2023.107318)
Supplement: Supplementary file 2 — Document S1. Figures S1–S8 and Data S1 and S2 [file mmc1.pdf]

iScience, Volume *26*

Supplemental information

The nuclear transport factor CSE1 drives macronuclear volume increase and macronuclear node coalescence in *Stentor coeruleus*

Rebecca M. McGillivary, Pranidhi Sood, Katherine Hammar, and Wallace F. Marshall

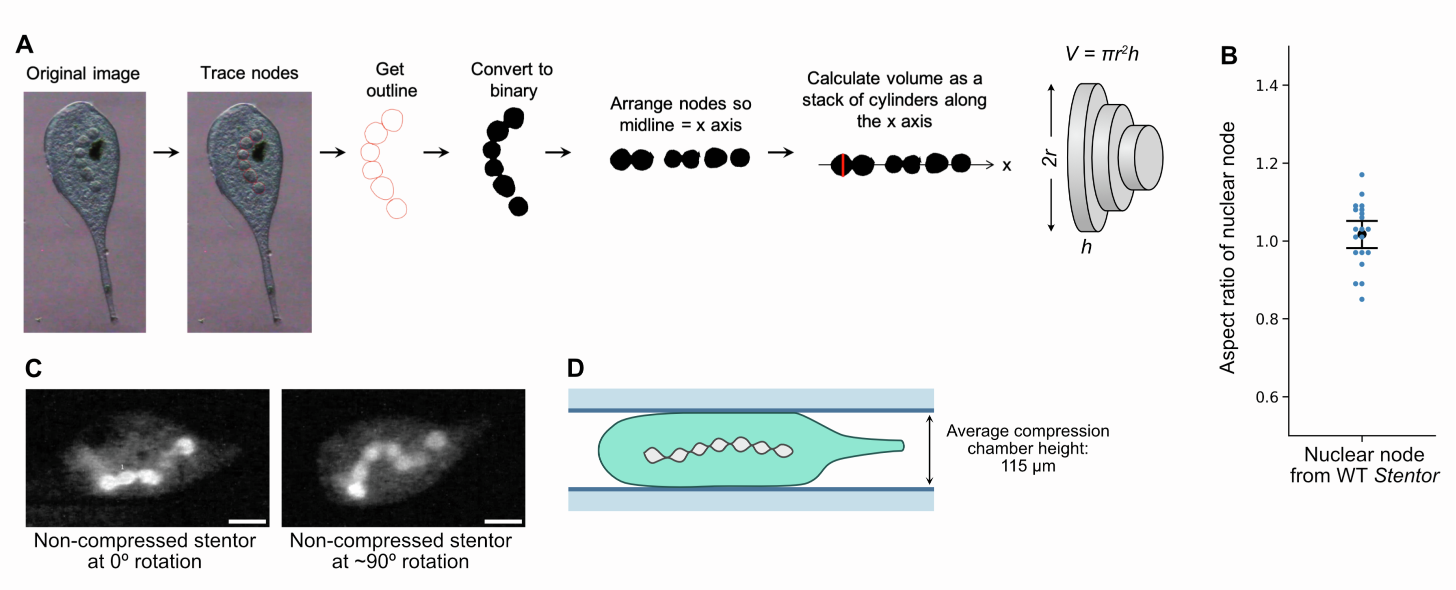

Supplemental Figure S1: Calculating macronuclear volumes during regeneration, related to Figure 1.

(A) Diagram of macronuclear volume calculation workflow. The macronuclear nodes are traced by hand. The traced outline is then separated from the image and filled in to create a binary image of the macronucleus. Then, the macronucleus image is split up and individual node images rotated so that the midline of the macronucleus is now a straight line along the x axis. The volume of the macronucleus is calculated by assuming rotational symmetry around the x axis to generate a series of cylinders of radius equal to that of the macronucleus relative to the axis at that position, and then adding up the volumes of cylinders along the macronucleus. The height of each cylinder is 1 pixel length along the x axis, and the diameter of each cylinder is the thickness of the macronucleus at that point. The red line illustrates a side view of one of these cylinders. The volume for each cylinder is calculated and added together to obtain the total macronuclear volume.

(B) Confirmation of rotational symmetry. Plot shows the average aspect ratio of *Stentor* nodes. Here we define aspect ratio in the YZ plane where Y is the diameter of the node in the plane of the image and Z is the diameter of the node along the vertical axis perpendicular to the image plane. The diameters of nuclear nodes were measured from movies of live, freely swimming *Stentors* swimming in a rotational manner (Video S1). The macronuclei of these stentors were stained with Hoechst 33342. The diameters of individual nodes were measured twice - once at a starting point we defined as 0° rotation, and a second time after the stentor completed approximately a 90° turn. The diameter at 0° rotation was divided by the diameter at 90° rotation to calculate the aspect ratio of the node's cross section. These aspect ratios for individual nodes are plotted as blue points. Rotational symmetry would predict an aspect ratio of 1.0. The average aspect ratio from our data is 1.02, and is shown by the black point on the plot. The error bars represent the 95% confidence interval. N=20.

(C) Images of one of the individual stentor cells used to compile panel B, depicting the approximately 90° turn used to calculate the aspect ratios of the macronuclear nodes. Scale bars = 75 pixels.

(D) Diagram showing the average chamber height (115 um) of the rotocompressor used while imaging regeneration. The diameters of the macronuclear nodes are drawn to scale. To calculate this height, we compared the diameters of 10 stentors pre- and post-compression. We assumed that the non-compressed stentors were rotationally symmetric, and measured the largest diameter across the cell. The cross section of a pre-compressed stentor would be a circle with radius r.

1) $A1 = \pi r^2$

For the compressed stentors we assumed there was no cellular volume change, so the area of the cross section of the compressed cell = the area of the cross section of the non-compressed cell.

2) $A1 = A2$

For the compressed stentors, their cross section would be an ellipse with small radius h and large radius R.

3) $A2 = \pi h R$

We measured both r and R, so we can arrange the equation to solve for h. The total chamber height would then be 2h.

4) $h = r^2 \div R$

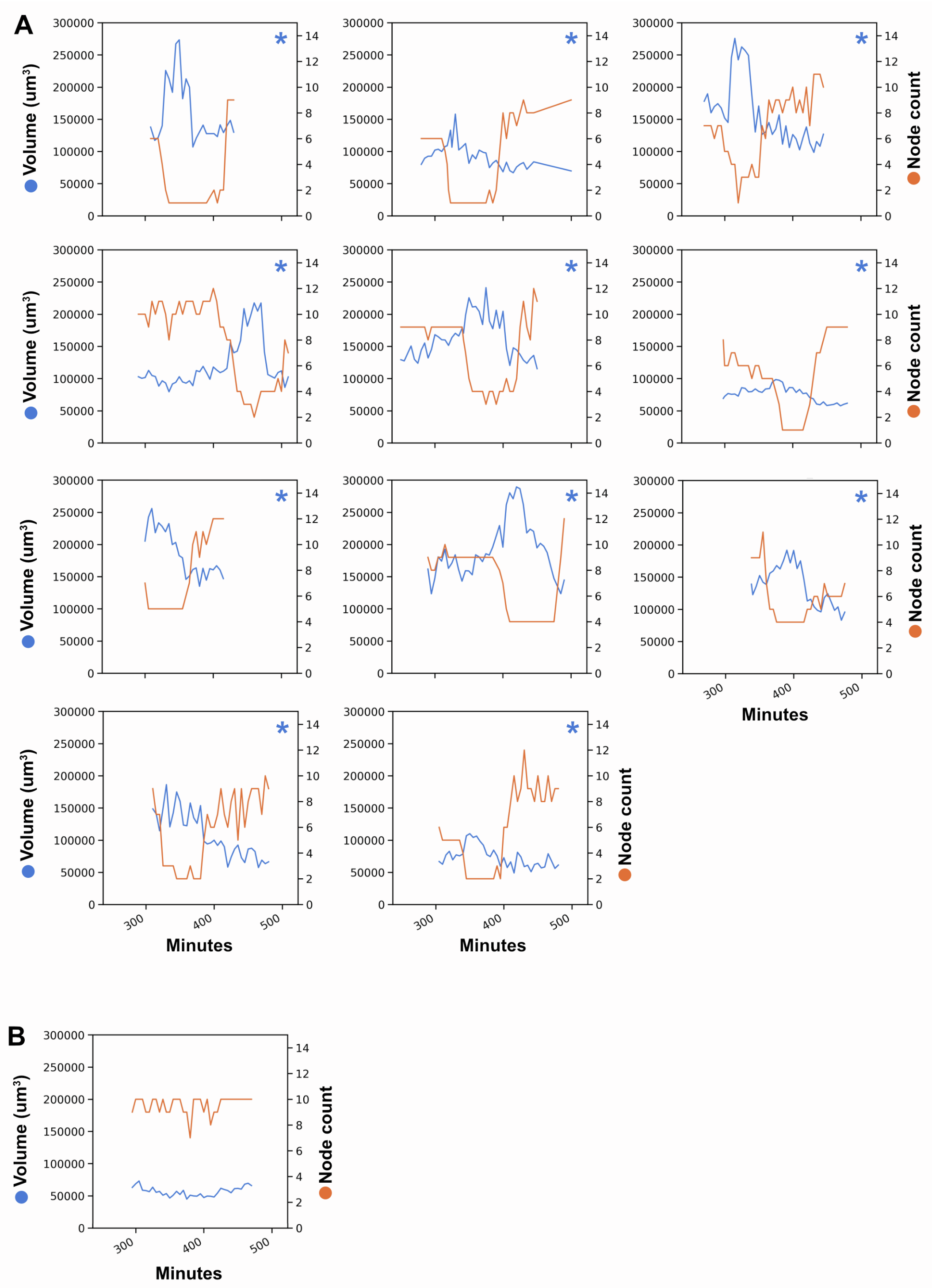

Supplemental Figure S2: Volume and node change during regeneration of *WT Stentor* , related to Figure 1.

(A) Plots of individual WT *Stentor* cells that underwent the macronuclear shape change cycle, showing the macronuclear volume and node number as a function of time. The total volume of the macronucleus at each time point is in blue, and the total number of nodes is in orange. The X axis is the time since sucrose shock in minutes. The overall macronuclear volumes of these timepoints were compared using a two-tailed Welch's t-Test. Stentors with statistically significant increases in macronuclear volume are marked with a blue asterisk (P<0.02). This was calculated by defining the timepoint ranges that encompass the highest quartile of node counts, and the lowest quartile of node counts, and then averaging the macronuclear volume over each of the two resulting sets of time points. This statistical test was applied individually to each cell, with an average of 9-12 time points for each quartile, as described in Methods.

(B) Plot of an individual WT *Stentor* cell that did not undergo the macronuclear shape change cycle. The total volume of the macronucleus at each time point is in blue, and the total number of nodes is in orange. The X axis is the time since sucrose shock in minutes.

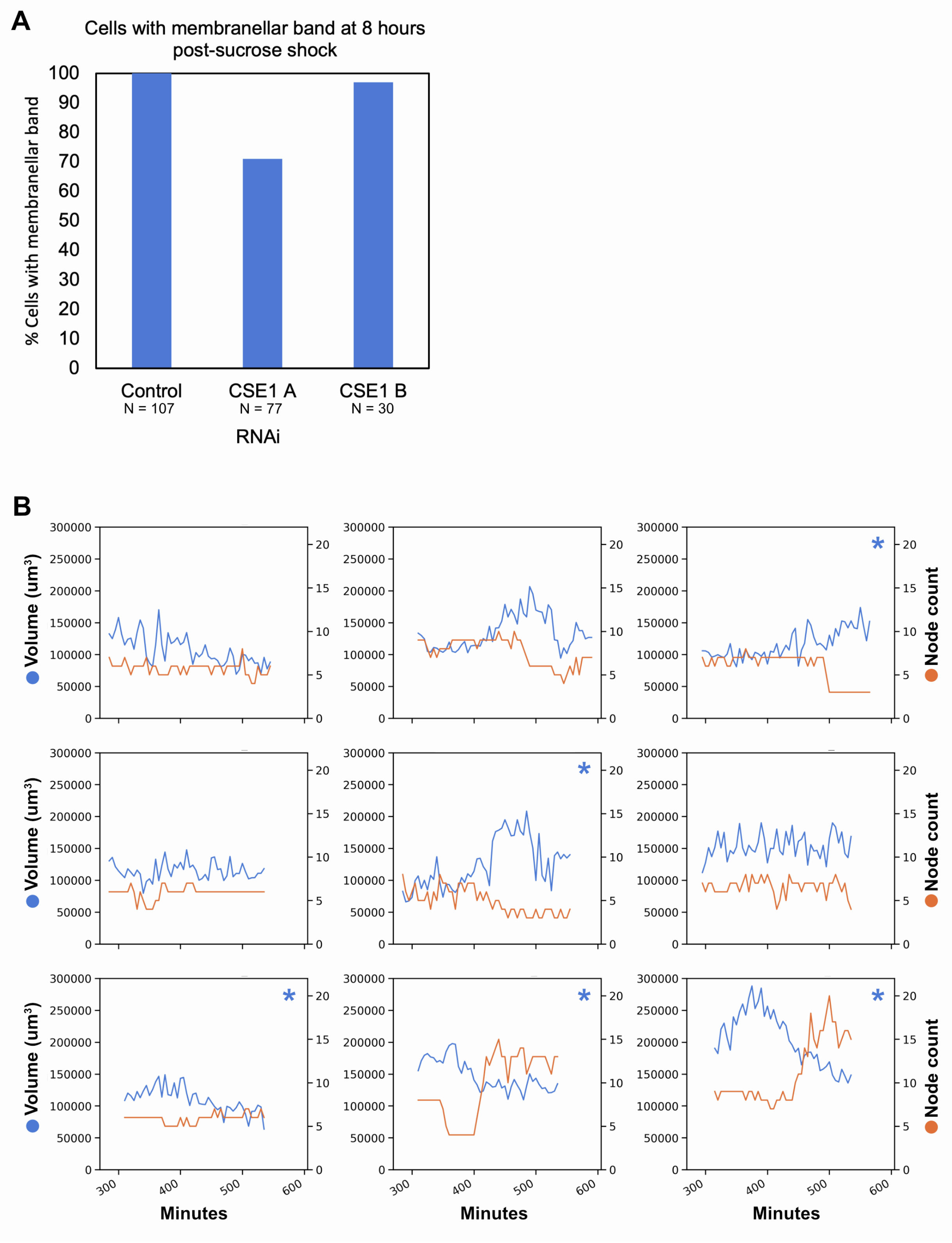

Supplemental Figure S3. Related to Figure 3. Regeneration of *CSE1(RNAi) Stentor*

(A) Plot showing the percentage of *Stentor* that re-grew membranellar bands 8 hours after sucrose shock. 100% of control *(LF4 RNAi) Stentor* had a membranellar band 8 hours after sucrose shock (N = 107). For *CSE1(RNAi) A Stentor,* 71% of cells regenerated a membranellar band 8 hours after sucrose shock (N = 77). For *CSE1(RNAi) B Stentor,* 97% of cells regenerated a membranellar band 8 hours after sucrose shock (N = 30).

(B) Plots of individual *CSE1(RNAi) B Stentor* volume and node number changes during regeneration. The total volume of the macronucleus over time is in blue, and the total number of nodes is in orange. Stentors with statistically significant increases in macronuclear volume are marked with a blue asterisk (P<0.05). Statistical testing was performed on individual cells as in Supplementary Figure S2.

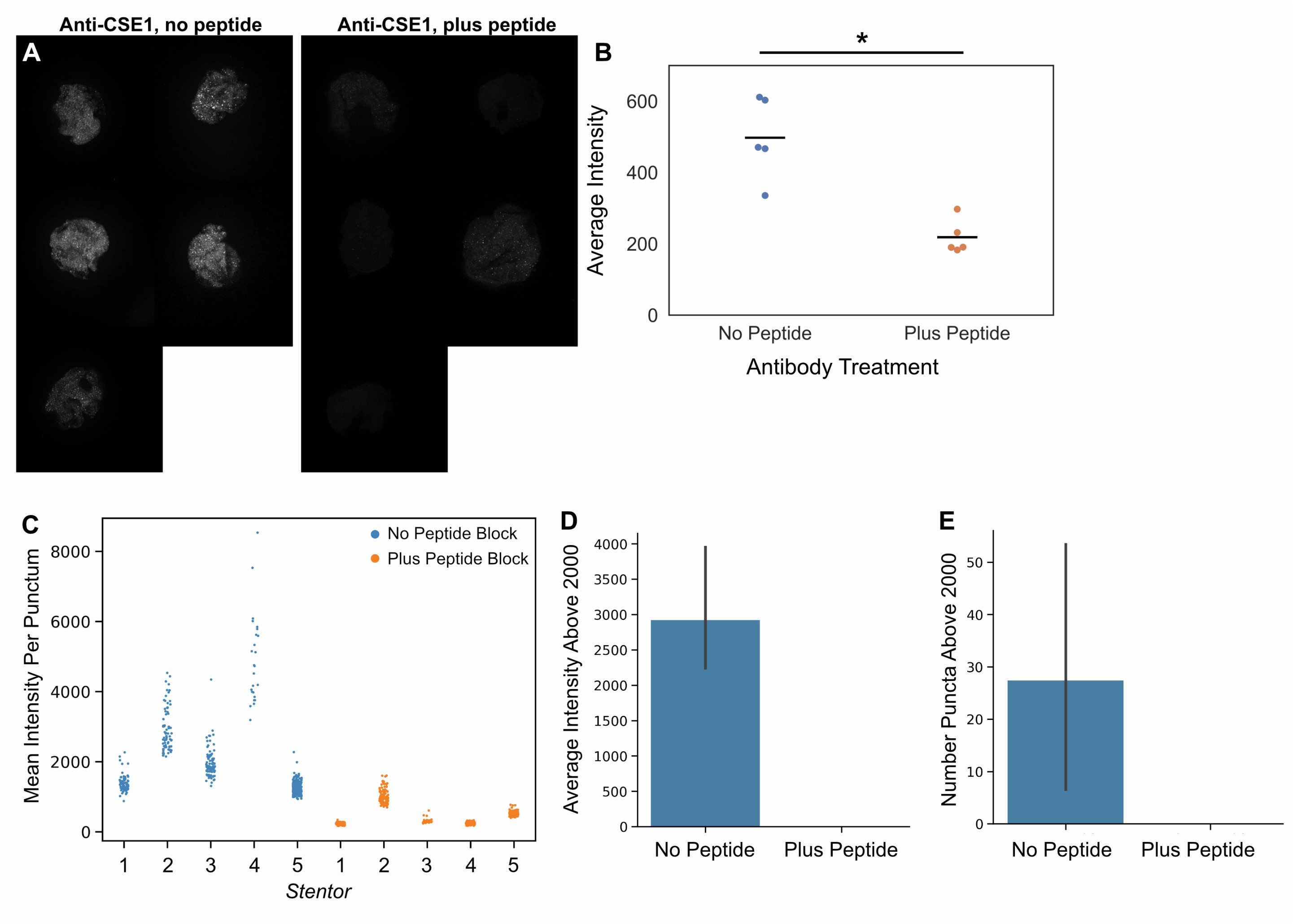

Supplemental Figure S4: Anti-CSE1 antibody peptide block control, related to Figure 3 and Figure 4.

(A) Peptide blocking control. Images of PFA fixed *Stentor* stained with anti-CSE1 either without or with pre-incubation with CSE1 peptide. Cells were imaged with a W1 spinning disk confocal.

(B) Plot showing the average CSE1 staining intensity in PFA fixed *Stentor* when the CSE1 antibody is either on its own or pre-incubated with CSE1 peptide. Without the peptide block, the average CSE1 intensity is 497 (n = 5). With peptide blocking the average CSE1 intensity drops in half to 218. *P < 0.01, Two-tailed t-Test.

(C) Puncta were automatically thresholded using the moments thresholding method. The average intensity of each punctum between the sizes of 0.75 μm – 10 μm was measured and plotted for each cell. Points in blue represent puncta from cells in (A) that did not have a peptide block. Points in orange represent cells in (A) that did have a peptide blocked antibody.

(D) Puncta that had an intensity below 2000 were discarded. The intensity of puncta above this cutoff were averaged for each cell. These were averaged together for each treatment and plotted with an error bar showing the 95% confidence interval. The average puncta intensity for cells without a peptide block is 2920. Cells with a peptide block do not have any puncta over an intensity of 2000.

(E) The number of puncta with intensities above 2000 were averaged for each cell and plotted with an error bar showing the 95% confidence interval. The average number of puncta above 2000 in cells without a peptide block is 27.4. Cells with a peptide block do not have any puncta over an intensity of 2000.

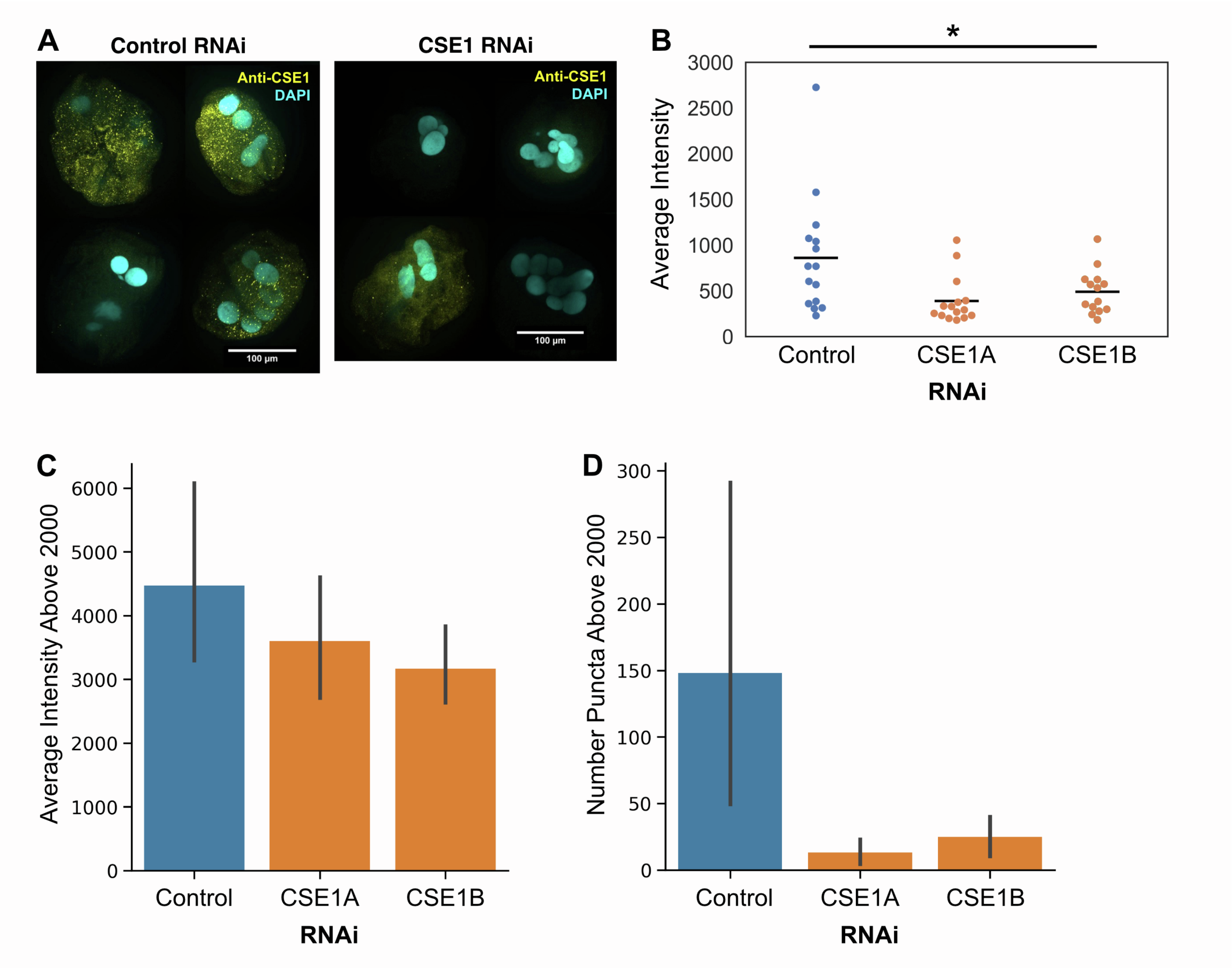

Supplemental Figure S5: Anti-CSE1 antibody RNAi control, related to Figure 3 and Figure 4.

(A) Images of PFA fixed *LF4(RNAi)* or *CSE1(RNAi) Stentor* stained with anti-CSE1. Cells were imaged with a W1 spinning disk confocal.

(B) Plot showing the average CSE1 staining intensity in PFA fixed *Stentor* treated with RNAi. In Control *LF4 (RNAi) Stentor*, the average intensity is 860. In *CSE1(RNAi) A Stentor*, the average intensity is 388. The average intensity of *CSE1(RNAi) B Stentor* is 489. *P < 0.05, One-tailed t-Test (individual P values 0.007 for Cse1(RNAi) A and 0.027 for CSE1(RNAi) B. Similar statistical results were obtained by a one-tailed Mann-Whitney U test (P values of 0.003 and 0.038 respectively). Note that the two RNAi constructs are from two different, non-overlapping parts of the CSE1 gene, and both cause a reduction in signal with the anti-CSE1 antibody.

(C) CSE1 puncta were segmented as in Supplementary Figure S4. The intensity of all puncta with intensities above 2000 were averaged for each cell. These intensities were averaged according to RNAi treatment and plotted with an error bar showing the 95% confidence interval. There is no significant difference between the intensity of CSE1 puncta in Control or *CSE1 RNAi Stentor.*

(D) The number of puncta with intensities above 2000 were counted for each cell. These counts were averaged according to RNAi treatment and plotted with an error bar showing the 95% confidence interval. Both *CSE1 (RNAi) A* and *CSE1 (RNAi) B Stentor* have significantly fewer CSE1 puncta.

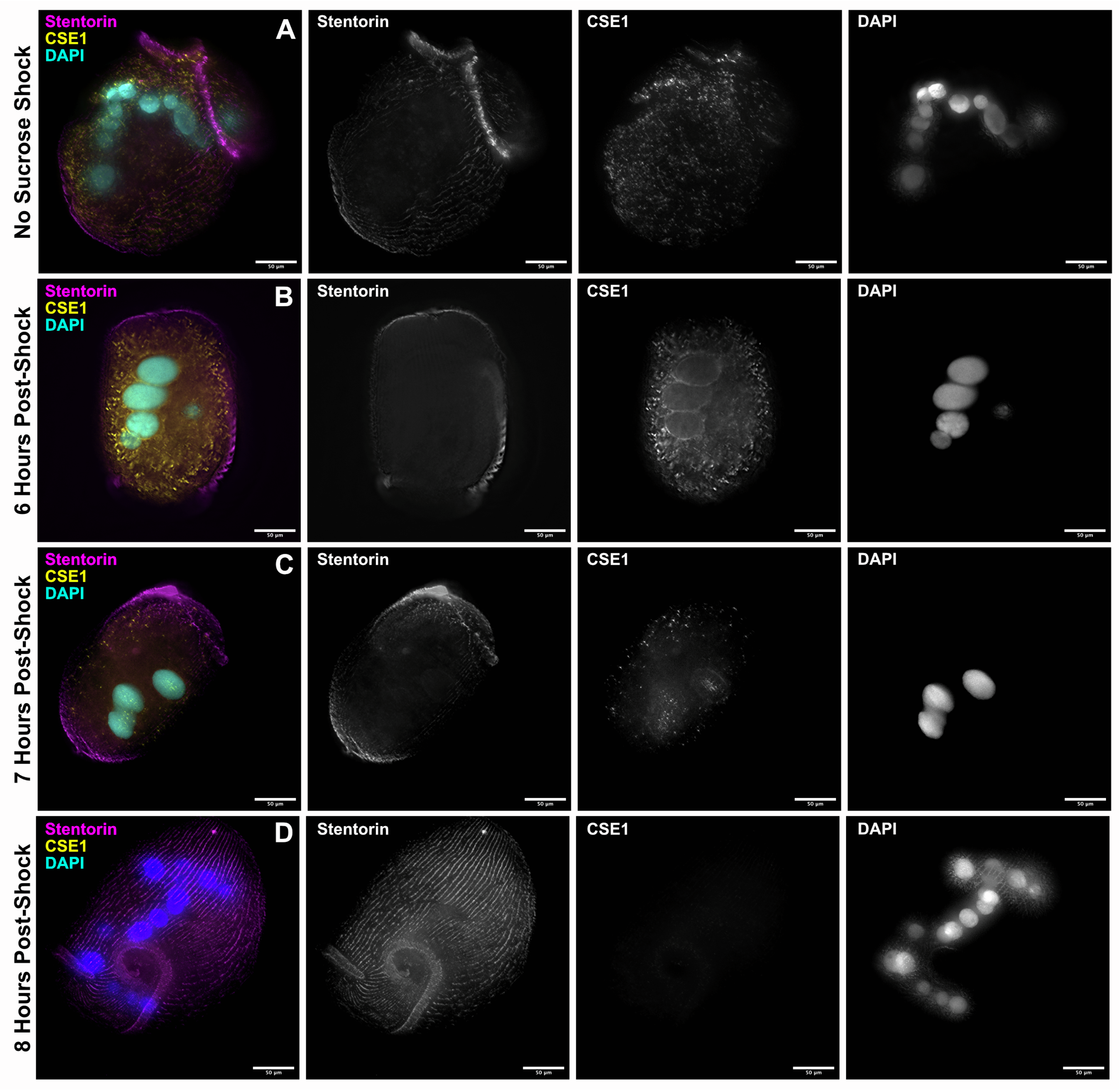

Supplemental Figure S6: Immunofluorescence of CSE1 in methanol fixed *Stentor* at different stages of regeneration, related to Figure 4.

All cells were imaged with a 20x air objective on a DeltaVision deconvolution microscope. Scale bars = 50 um.

(A) *Stentor* that have not undergone sucrose shock have cytoplasmic puncta of CSE1.

(B) Six hours post sucrose shock is generally the time at which the macronucleus begins coalescence. At this time we observed CSE1 staining around the periphery of the macronucleus.

(C) At 7 hours post sucrose shock the macronucleus in this cell is coalesced. At this time we observe CSE1 puncta inside of the macronucleus.

(D) At 8 hours post sucrose shock, the total CSE1 signal is greatly decreased relative to pre-sucrose shock stentors. Panels A-D are scaled to the same range of intensities.

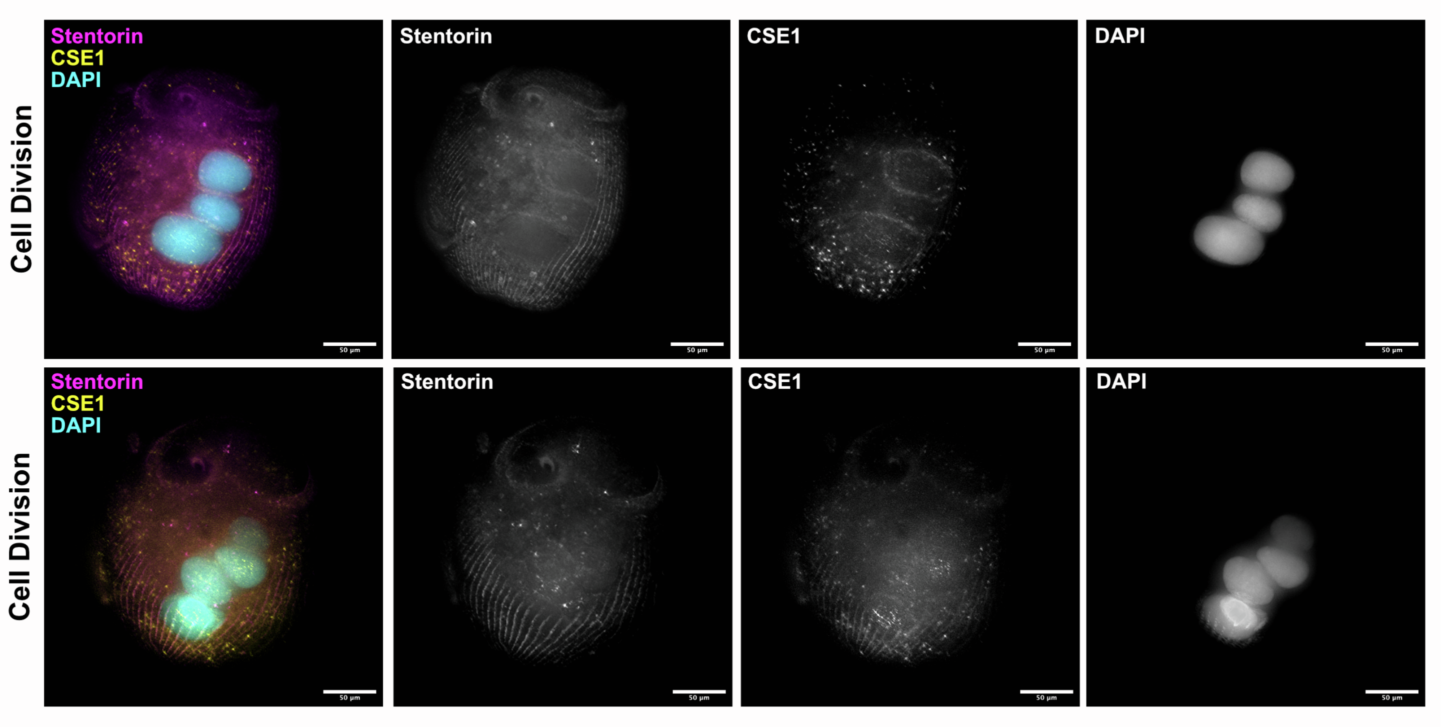

Supplemental Figure S7: Immunofluorescence of CSE1 in dividing *Stentor* fixed with methanol, related to Figure 4. We imaged cells undergoing nuclear coalescence during cell division, rather than during regeneration. *Stentor* late in cell division are readily identifiable in culture because they have a visible membranellar band developing on the side of the cell, as well as the original membranellar band at the anterior end. Cells were imaged with a 20x air objective on a DeltaVision deconvolution microscope. Scale bars = 50 um. In cells with coalesced macronuclei we observe CSE1 around the periphery of the macronucleus, as well as punctate CSE1 staining in the interior of the macronucleus, similar to what we observed in cells undergoing nuclear coalescence during regeneration. The two rows of images show two different cells undergoing division.

RNAi Name	Target Gene	Position within coding region	Primer Sequences
CSE1A	CSE1 (g28374)	1247 - 2084	Forward<br>AACTGGAAGCAAATGGATGC<br>Reverse<br>AGCCATAGATGAGGCATTGG
CSE1B	CSE1 (g28374)	2089-2841	Forward<br>TGCCTCATCTATGGCTCCTC<br>Reverse<br>TTGAAGAGTTGGGCGTATCG
LF4	LF4	8-884	Forward<br>CAGAATACCGTTTGATATCAAAAAAAGGTGAAGG<br>Reverse<br>CTACTTGCCTCATCAACAAAATCTTTGAAATAAGG
Mob1	Mob1	40-672	Forward<br>AAGAAGCGAATTGAAAAAGGCCAGC<br>Reverse<br>GTTACCAGAAGCTTCTCTTTCCATTCTTTCC

Supplemental Figure S8: List of RNAi constructs and primers, related to Figure 3.

A

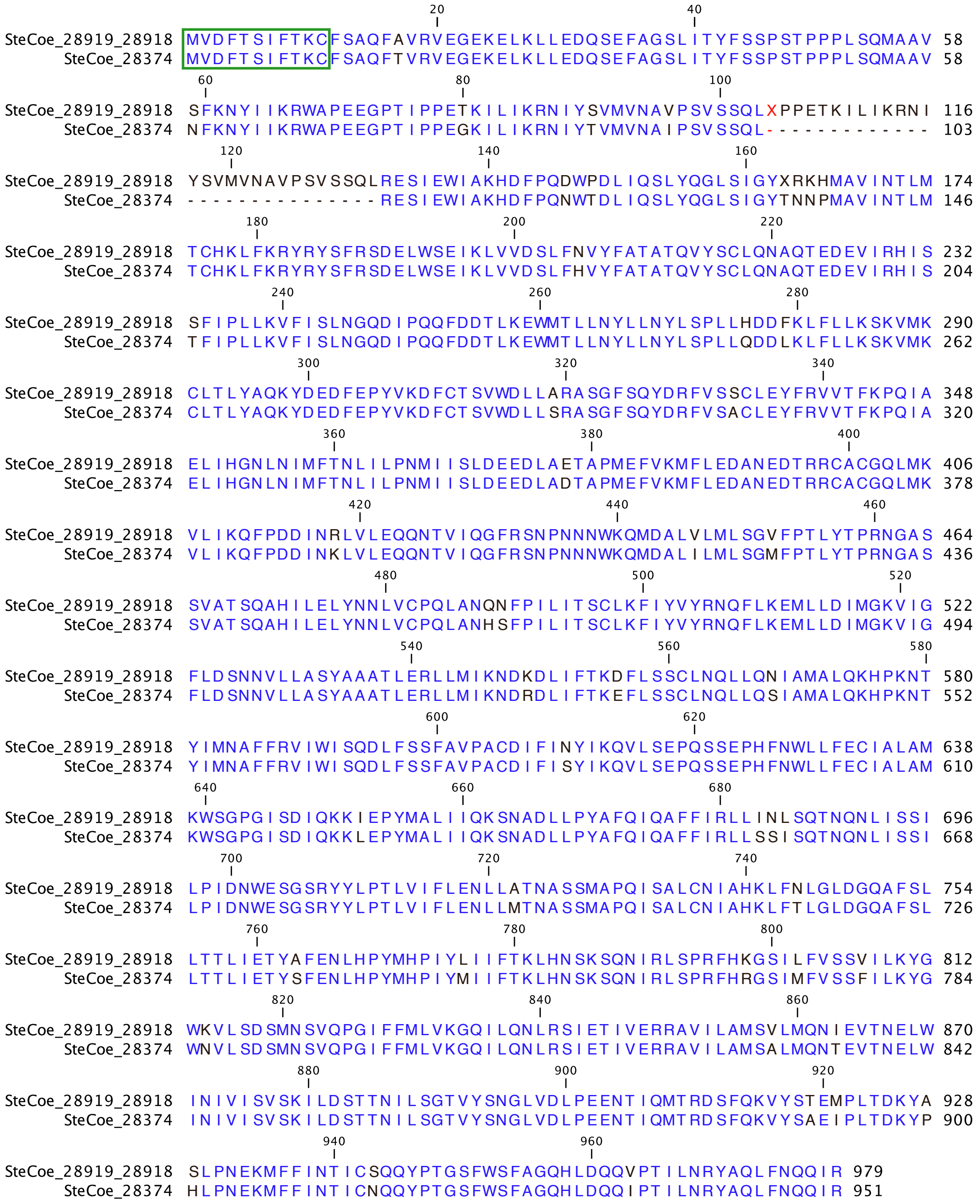
SteCoe_28919_28918 MVDFTSIFTKCFSAQFAVRVEGEKELKLLEDQSEFAGSLITYFSSPSTPPPLSQMAAV 58
SteCoe_28374 MVDFTSIFTKCFSAQFTVRVEGEKELKLLEDQSEFAGSLITYFSSPSTPPPLSQMAAV 58
SteCoe_28919_28918 SFKNYIIKRWAPEEGPTIPPETKILIKRNIYSVMVNAVPSVSSQLXPPETKILIKRNI 116
SteCoe_28374 NFKNYIIKRWAPEEGPTIPPEGKILIKRNIYTVMVNAIPSVSSQL------------- 103
SteCoe_28919_28918 YSVMVNAVPSVSSQLRESIEWIAKHDFPQDWPDLIQSLYQGLSIGYXRKHMAVINTLM 174
SteCoe_28374 ---------------RESIEWIAKHDFPQNWTDLIQSLYQGLSIGYTNNPMAVINTLM 146
SteCoe_28919_28918 TCHKLFKRYRYSFRSDELWSEIKLVVDSLFNVYFATATQVYSCLQNAQTEDEVIRHIS 232
SteCoe_28374 TCHKLFKRYRYSFRSDELWSEIKLVVDSLFHVYFATATQVYSCLQNAQTEDEVIRHIS 204
SteCoe_28919_28918 SFIPLLKVFISLNGQDIPQQFDDTLKEWMTLLNYLLNYLSPLLHDDFKLFLLKSKVMK 290
SteCoe_28374 TFIPLLKVFISLNGQDIPQQFDDTLKEWMTLLNYLLNYLSPLLQDDLKLFLLKSKVMK 262
SteCoe_28919_28918 CLTLYAQKYDEDFEPYVKDFCTSVWDLLARASGFSQYDRFVSSCLEYFRVVTFKPQIA 348
SteCoe_28374 CLTLYAQKYDEDFEPYVKDFCTSVWDLLSRASGFSQYDRFVSACLEYFRVVTFKPQIA 320
SteCoe_28919_28918 ELIHGNLNIMFTNLILPNMIISLDEEDLAETAPMEFVKMFLEDANEDTRRCACGQLMK 406
SteCoe_28374 ELIHGNLNIMFTNLILPNMIISLDEEDLADTAPMEFVKMFLEDANEDTRRCACGQLMK 378
SteCoe_28919_28918 VLIKQFPDDINRLVLEQQNTVIQGFRSNPNNNWKQMDALVLMLSGVFPTLYTPRNGAS 464
SteCoe_28374 VLIKQFPDDINKLVLEQQNTVIQGFRSNPNNNWKQMDALILMLSGMFPTLYTPRNGAS 436
SteCoe_28919_28918 SVATSQAHILELYNNLVCPQLANQNFPILITSCLKFIYVYRNQFLKEMLLDIMGKVIG 522
SteCoe_28374 SVATSQAHILELYNNLVCPQLANHSFPILITSCLKFIYVYRNQFLKEMLLDIMGKVIG 494
SteCoe_28919_28918 FLDSNNVLLASYAAATLERLLMIKNDKDLIFTKDFLSSCLNQLLQNIAMALQKHPKNT 580
SteCoe_28374 FLDSNNVLLASYAAATLERLLMIKNDRDLIFTKEFLSSCLNQLLQSIAMALQKHPKNT 552
SteCoe_28919_28918 YIMNAFFRVIWISQDLFSSFAVPACDIFINYIKQVLSEPQSSEPHFNWLLFECIALAM 638
SteCoe_28374 YIMNAFFRVIWISQDLFSSFAVPACDIFISYIKQVLSEPQSSEPHFNWLLFECIALAM 610
SteCoe_28919_28918 KWSGPGISDIQKKIEPYMALIIQKSNADLLPYAFQIQAFFIRLLINLSQTNQNLISSI 696
SteCoe_28374 KWSGPGISDIQKKLEPYMALIIQKSNADLLPYAFQIQAFFIRLLSSISQTNQNLISSI 668
SteCoe_28919_28918 LPIDNWESGSRYYLPTLVIFLENLLATNASSMAPQISALCNIAHKLFNLGLDGQAFSL 754
SteCoe_28374 LPIDNWESGSRYYLPTLVIFLENLLMTNASSMAPQISALCNIAHKLFTLGLDGQAFSL 726
SteCoe_28919_28918 LTTLIETYAFENLHPYMHPIYLIIFTKLHNSKSQNIRLSPRFHKGSILFVSSVILKYG 812
SteCoe_28374 LTTLIETYSFENLHPYMHPIYMIIFTKLHNSKSQNIRLSPRFHRGSIMFVSSFILKYG 784
SteCoe_28919_28918 WKVLSDSMNSVQPGIFFMLVKGQILQNLRSIETIVERRAVILAMSVLMQNIEVTNELW 870
SteCoe_28374 WNVLSDSMNSVQPGIFFMLVKGQILQNLRSIETIVERRAVILAMSALMQNTEVTNELW 842
SteCoe_28919_28918 INIVISVSKILDSTTNILSGTVYSNGLVDLPEENTIQMTRDSFQKVYSTEMPLTDKYA 928
SteCoe_28374 INIVISVSKILDSTTNILSGTVYSNGLVDLPEENTIQMTRDSFQKVYSAEIPLTDKYP 900
SteCoe_28919_28918 SLPNEKMFFINTICSQQYPTGSFWSFAGQHLDQQVPTILNRYAQLFNQQIR 979
SteCoe_28374 HLPNEKMFFINTICNQQYPTGSFWSFAGQHLDQQIPTILNRYAQLFNQQIR 951

B

```
                                20                  40                  60
CSE1_A_28918 AACTGGAAACAAATGGATGCATTAGTACTTATGCTTTCTGGGGTATTTCCTACACTTTATACT 63
CSE1_A_28374 AACTGGAAGCAAATGGATGCTTTAATACTTATGCTTTCTGGGATGTTTCCAACTTTATACACT 63
                             80                  100                 120
CSE1_A_28918 CCAAGAAATGGTGCATCATCAGTAGCGACATCACAAGCGCATATACTTGAACTTTATAACAAT 126
CSE1_A_28374 CCAAGAAATGGGGCTTCATCAGTAGCGACATCACAAGCACATATTCTTGAACTTTATAACAAT 126
                          140                 160                 180
CSE1_A_28918 TTAGTCTGTCCTCAATTAGCTAATCAGAATTTCCCTATACTTATTACAAGTTGTCTTAAGTTT 189
CSE1_A_28374 TTGGTCTGTCCTCAACTTGCTAATCATAGTTTCCCTATACTAATAACAAGTTGTCTTAAGTTT 189
                       200                 220                 240
CSE1_A_28918 ATTTACGTCTACAGAAACCAGTTTTTAAAAGAAATGCTTTTGGACATAATGGGAAAAGTTATA 252
CSE1_A_28374 ATTTATGTCTACAGAAATCAGTTTTTAAAAGAAATGCTATTAGATATCATGGGAAAAGTTATT 252
                    260                 280                 300
CSE1_A_28918 GGGTTTTTAGACAGTAACAATGTTTTATTAGCAAGTTATGCTGCGGCTACACTTGAAAGGCTG 315
CSE1_A_28374 GGGTTCTTAGATAGTAACAACGTATTATTAGCCAGTTATGCTGCAGCTACTCTTGAAAGACTT 315
                 320                 340                 360
CSE1_A_28918 CTTATGATAAAAAATGATAAGGATTTGATTTTCACTAAAGACTTCCTGTCTTCATGTTTGAAC 378
CSE1_A_28374 CTTATGATAAAAAACGACCGGGATCTTATTTTCACTAAAGAGTTCCTATCATCATGTTTAAAT 378
              380                 400                 420                 440
CSE1_A_28918 CAATTATTACAAAACATTGCTATGGCTCTTCAGAAACACCCTAAAAACACTTATATTATGAAT 441
CSE1_A_28374 CAATTATTACAAAGTATCGCTATGGCTCTTCAGAAACACCCAAAAAACACTTATATTATGAAT 441
                               460                 480                 500
CSE1_A_28918 GCATTTTTCAGAGTCATCTGGATATCCCAAGACCTGTTTTCAAGTTTTGCAGTCCCAGCTTGT 504
CSE1_A_28374 GCATTTTTTAGGGTTATTTGGATATCCCAAGATTTGTTTTCAAGTTTTGCAGTCCCAGCTTGT 504
                            520                 540                 560
CSE1_A_28918 GACATTTTTATCAATTACATCAAACAAGTTCTTTCTGAGCCTCAGAGTTCAGAACCTCATTTC 567
CSE1_A_28374 GACATTTTTATCAGTTATATCAAGCAAGTACTTTCTGAGCCTCAAAGCTCAGAACCTCATTTC 567
                         580                 600                 620
CSE1_A_28918 AATTGGTTGCTCTTTGAGTGTATCGCCCTAGCTATGAAATGGTCAGGGCCTGGGATTTCTGAT 630
CSE1_A_28374 AACTGGTTACTTTTTGAATGTATTGCTTTAGCTATGAAATGGTCTGGGCCTGGGATTTCTGAT 630
                      640                 660                 680
CSE1_A_28918 ATACAGAAAAAAATCGAACCTTACATGGCTTTAATAATCCAAAAATCCAACGCAGATCTTTTA 693
CSE1_A_28374 ATCCAGAAAAAACTCGAACCTTATATGGCTCTGATAATCCAAAAGTCCAACGCAGACCTTTTG 693
                   700                 720                 740
CSE1_A_28918 CCATATGCTTTTCAGATCCAAGCTTTTTTCATAAGACTCCTGATTAATCTTTCACAAACCAAT 756
CSE1_A_28374 CCTTATGCTTTTCAAATCCAAGCTTTTTTCATAAGACTTTTGAGTAGTATTTCACAAACTAAT 756
                760                 780                 800
CSE1_A_28918 CAAAATCTTATTTCCTCCATACTCCCTATAGACAACTGGGAATCAGGAAGTAGATATTATTTA 819
CSE1_A_28374 CAAAATCTTATTTCCTCAATACTCCCTATAGACAATTGGGAATCAGGTAGTAGGTATTATTTA 819
             820                 840                 860
CSE1_A_28918 CCAACACTTGTTATTTTCTTAGAAAACCTTTTAGCAACTAATGCATCTTCTATGGCT 876
CSE1_A_28374 CCAACACTTGTGATTTTCTTAGAAAACCTTTTAATGACCAATGCCTCATCTATGGCT 876
```

C

```
                                   20                       40                       60
CSE1_B_28918  TGCATCTTCTATGGCTCCTCAAATATCTGCTCTATGTAATATTGCCCATAAACTTTTTAATTT  63
CSE1_B_28374  TGCCTCATCTATGGCTCCTCAGATTTCAGCATTATGTAACATTGCCCATAAACTTTTTACTTT  63
                                80                      100                      120
CSE1_B_28918  AGGATTAGATGGTCAAGCATTTTCCTTACTTACAACGTTAATTGAAACTTATGCTTTTGAAAA  126
CSE1_B_28374  AGGATTAGATGGTCAAGCCTTTTCTTTACTTACAACTCTAATCGAAACTTATTCTTTTGAGAA  126
                             140                      160                      180
CSE1_B_28918  TTTACATCCTTATATGCACCCGATTTATCTGATAATTTTTACTAAATTGCATAATTCTAAGTC  189
CSE1_B_28374  TTTACACCCTTATATGCACCCGATTTATATGATAATTTTCACTAAACTGCACAATTCTAAGTC  189
                          200                      220                      240
CSE1_B_28918  TCAGAATATTAGACTTAGTCCCCGTTTCCATAAAGGGTCGATATTATTTGTCAGTTCGGTTAT  252
CSE1_B_28374  TCAGAATATCAGACTTAGTCCACGTTTTCATAGGGGGTCGATAATGTTTGTTAGTTCATTTAT  252
                       260                      280                      300
CSE1_B_28918  ATTGAAATATGGATGGAAAGTCTTATCTGATAGTATGAATTCAGTTCAACCAGGGATATTTTT  315
CSE1_B_28374  ATTGAAATATGGATGGAATGTTCTTTCTGATAGTATGAACTCAGTTCAACCAGGGATATTTTT  315
                    320                      340                      360
CSE1_B_28918  TATGCTAGTTAAAGGGCAGATATTACAAAATTTGAGGTCTATTGAGACGATTGTTGAAAGAAG  378
CSE1_B_28374  CATGTTGGTTAAAGGGCAGATATTGCAAAATTTAAGGTCTATTGAAACAATTGTTGAAAGAAG  378
               380                      400                      420                      440
CSE1_B_28918  AGCTGTGATTTTAGCTATGAGTGTGCTTATGCAAAATATTGAAGTTACTAATGAATTGTGGAT  441
CSE1_B_28374  AGCTGTGATTTTAGCTATGAGTGCGCTTATGCAGAATACTGAGGTTACTAATGAACTATGGAT  441
                                  460                      480                      500
CSE1_B_28918  TAATATTGTTATTAGTGTTAGTAAGATACTTGATAGTACAACGAATATACTTAGTGGGACGGT  504
CSE1_B_28374  TAATATTGTTATTAGTGTTAGTAAAATACTTGATAGTACAACAAATATACTTAGTGGGACAGT  504
                               520                      540                      560
CSE1_B_28918  GTATTCTAATGGACTTGTTGACTTACCTGAAGAAAACACTATACAAATGACACGAGACTCCTT  567
CSE1_B_28374  GTATTCTAATGGACTTGTTGACTTGCCTGAAGAAAACACGATACAAATGACTAGAGATTCCTT  567
                            580                      600                      620
CSE1_B_28918  CCAAAAGGTCTACAGCACTGAAATGCCTTTAACAGACAAATACGCAAGCCTTCCTAATGAAAA  630
CSE1_B_28374  CCAAAAAGTATACAGCGCTGAAATCCCTTTAACAGACAAATACCCACACCTTCCTAACGAAAA  630
                         640                      660                      680
CSE1_B_28918  AATGTTCTTCATCAACACAATCTGTAGCCAGCAATACCCAACTGGATCCTTCTGGAGCTTTGC  693
CSE1_B_28374  AATGTTTTTCATCAACACAATCTGTAACCAGCAATACCCAACAGGATCTTTCTGGAGCTTTGC  693
                      700                      720                      740
CSE1_B_28918  CGGGCAACATCTAGACCAACAAGTTCCAACAATCTTAAATCGATATGCCCAGCTCTTCAA  753
CSE1_B_28374  CGGTCAACATTTAGATCAACAAATTCCAACAATCCTCAATCGATACGCCCAACTCTTCAA  753
```

Data S1: Alignment of *Stentor's* CSE1 genes, related to Figure 2.

(A) Pairwise alignment of protein sequences of *Stentor's* two CSE1 orthologs. Gene 28374 is the CSE1 gene that RNAi constructs were made against. Gene 28919+28918 is a slightly longer paralog that encodes a protein 92% identical to that encoded by gene 28374. Identical residues are colored in blue. The green box shows the peptide sequence that the CSE1 antibody was raised against. Both paralogs have the identical amino acid sequence in this region.

(B) Pairwise alignment of the DNA sequence targeted by CSE1 RNAi Construct A (SteCoe_28374) and the corresponding DNA sequence of SteCoe_28918. These sequences are 89% identical (780/878 nucleotides). Identical nucleotides are colored in blue.

(C) Pairwise alignment of the DNA sequence targeted by CSE1 RNAi Construct B (SteCoe_28374) and the corresponding DNA sequence of SteCoe_28918. These sequences are 90% identical (678/754 nucleotides). Identical nucleotides are colored in blue.

```
SteCoe_g28919+8    1 ----MVDFTSI---FTKCFSAQFAVRVEGEKELKLLEDQSEFAGSLITYFSSPSTPPPLS
SteCoe_g28374      1 ----MVDFTSI---FTKCFSAQFTVRVEGEKELKLLEDQSEFAGSLITYFSSPSTPPPLS
SacCer_CSE1        1 ----MSDLETVAKFLAESVIASTA--KTSERNLRQLETQDGFGLTLLHVIASTNLPLSTR
DroMel_CSE1        1 MEVTEANLQLLAGYLQQTLSADPNVRRPAEKLLESTELQQNYPILLLNLIDKAQMDMTTR
HomSap_CAS         1 MELSDANLQTLTEYLKKTLDPDPAIRRPAEKFLESVEGNQNYPLLLLTLLEKSQ-DNVIK
MusMus_XPO2        1 MELSDANLQTLTEYLKKTLDPDPAIRRPAEKFLESVEGNQNYPLLLLTLLEKSQ-DNVIK

SteCoe_g28919+8   54 QMAAVSFKNYIIKRW---APEEGPTIPPETKILIKRNIYSVMVNAVPSVSSQLXPPETKI
SteCoe_g28374     54 QMAAVNFKNYIIKRW---APEEGPT----------------------------IPPEGKI
SacCer_CSE1       55 LAGALFFKNFIKRKW---VDENGNH----------------------------LLPANNVE
DroMel_CSE1       61 VAGAIAFKNYIKRNWAAHLDSDGPD----------------------------RIHESDRN
HomSap_CAS        60 VCASVTFKNYIKRNW--RIVEDEPN----------------------------KICEADRV
MusMus_XPO2       60 VCASVTFKNYIKRNW--RIVEDEPN----------------------------KICEADRV

SteCoe_g28919+8  111 LIKRNIYSVMVNAVPSVSSQLRESIEWIAKHDFPQDWPDLIQSLYQGLSIGYXRKHMAVI
SteCoe_g28374     83 LIKRNIYTVMVNAIPSVSSQLRESIEWIAKHDFPQNWTDLIQSLYQGLSIGYTNNPMAVI
SacCer_CSE1       85 LIKKEIVPLMISLPNNLQVQIGEAISSIADSDFPDRWPTLLSDLASRLSND----DMVTN
DroMel_CSE1       94 TIKTLIVTLMLHSPVALQKQLSDAVSIIGKYDFPKKWPQLIDEMVERFASG----DFNVI
HomSap_CAS        91 AIKANIVHLMLSSPEQIQKQLSDAISIIGREDFPQKWPDLLTEMVNRFQSG----DFHVI
MusMus_XPO2       91 AIKANIVHLMLSSPEQIQKQLSDAISIIGREDFPQKWPDLLTEMVNRFQSG----DFHVI

SteCoe_g28919+8  171 N-TLMTCHKLFKRYRYSFRSDELWSEIKLVVDSL---FNVYFATATQVYSCLQNAQTEDE
SteCoe_g28374    143 N-TLMTCHKLFKRYRYSFRSDELWSEIKLVVDSL---FHVYFATATQVYSCLQNAQTEDE
SacCer_CSE1      141 KGVLTVAHSIFKRWRPLFRSDELFLEIKLVLDVFTAPFLNLLKTVDEQITANENNKASLN
DroMel_CSE1      150 NGVLQTAHSLFKRYRYEFKSQALWEEIKFVLDRMAKPLTDLLQATMQLTKVHENNAGALK
HomSap_CAS       147 NGVLRTAHSLFKRYRHEFKSNELWTEIKLVLDAFALPLTNLFKATIELCSTHANDASALR
MusMus_XPO2      147 NGVLRTAHSLFKRYRHEFKSNELWTEIKLVLDAFALPLTNLFKATIELCSTHANDASALR

SteCoe_g28919+8  227 VIRHISSFIPLLKVFISLNGQDIPQQFDDTLKEWMTLLNYLLNYLSPLLHDDFK------
SteCoe_g28374    199 VIRHISTFIPLLKVFISLNGQDIPQQFDDTLKEWMTLLNYLLNYLSPLLQDDLK------
SacCer_CSE1      201 IL--FDVLLVLIKLYYDFNCQDIPEFFEDNIQVGMGIFHKYLSYSNPLLEDPDETEHASV
DroMel_CSE1      210 VI--YGSLVLVNKVFFSLNSQDLPEFFEDNINTWMGAFIQQLAADVPSLRTADD-EDAGV
HomSap_CAS       207 IL--FSSLILISKLFYSLNFQDLPEFFEDNMETWMNNFHTLLTLDNKLLQTDDE-EEAGL
MusMus_XPO2      207 IL--FSSLILISKLFYSLNFQDLPEFFEDNMETWMNNFHTLLTLDNKLLQTDDE-EEAGL

SteCoe_g28919+8  281 LFLLKSKVMKCLTLYAQKYDEDFEPYVKDFCTSVWDLLARASGFSQYDRFVSSCLEYFRV
SteCoe_g28374    253 LFLLKSKVMKCLTLYAQKYDEDFEPYVKDFCTSVWDLLSRASGFSQYDRFVSACLEYFRV
SacCer_CSE1      259 LIKVKSSIQELVQLYTTRYEDVFGPMINEFIQITWNLLTSISNQPKYDILVSKSLSFLTA
DroMel_CSE1      267 LEHLRAQVCENICLYAKKYDEEFKPFMEQFVTAVWELLVKTSLHTKYDSLVSHALQFLSV
HomSap_CAS       264 LELLKSQICDNAALYAQKYDEEFQRYLPRFVTAIWNLLVTTGQEVKYDLLVSNAIQFLAS
MusMus_XPO2      264 LELLKSQICDNAALYAQKYDEEFQRYLPRFVTAIWNLLVTTGREVKYDLLVSNAIQFLAS

SteCoe_g28919+8  341 VTFKPQIAELIHGN--LNIMFTNLILPNMIISLDEEDLAETAPMEFVKMFLEDANEDTRR
SteCoe_g28374    313 VTFKPQIAELIHGN--LNIMFTNLILPNMIISLDEEDLADTAPMEFVKMFLEDANEDTRR
SacCer_CSE1      319 VTRIPKYFEIFNNESAMNNITEQIILPNVTLREEDVELFEDDPIEYIRRDLEGSDTDTRR
DroMel_CSE1      327 VADRQHYQSIFENPEILAQICDKVVIPNLDIRPSDEEIFEDSPEEYIRRDIEGSDIDTRR
HomSap_CAS       324 VCERPHYKNLFEDQNTLTSICEKVIVPNMEFRAADEEAFEDNSEEYIRRDLEGSDIDTRR
MusMus_XPO2      324 VCERPHYKNLFEDQNTLTSICEKVIVPNMEFRAADEEAFEDNSEEYIRRDLEGSDIDTRR

SteCoe_g28919+8  399 CACGQLMKVLIKQFPDDINRLVLEQQNTVIQGFRSNPNNNWKQMDALVLMLSGVFPTLYT
SteCoe_g28374    371 CACGQLMKVLIKQFPDDINKLVLEQQNTVIQGFRSNPNNNWKQMDALILMLSGMFPTLYT
SacCer_CSE1      379 RACTDFLKELKEKNEVLVTNIFLAHMKGFVDQYMSDPSKNWKFKDLYIYLFTALAINGNI
```

```
DroMel_CSE1       387 RAACDLVKTLSINFEQKIFGIFGQYLERLLTKYKENPATNWRSKDTAIYLVTSWASRGGT
HomSap_CAS        384 RAACDLVRGLCKFFEGPVTGIFSGYVNSMLQEYAKNPSVNWKHKDAAIYLVTSLASKAQT
MusMus_XPO2       384 RAACDLVRGLCKFFEGPVTGIFSGYVNSMLQEYAKNPSVNWKHKDAAIYLVTSLASKAQT

SteCoe_g28919+8   459 PRNGASSVATSQAHILELYNNLVCPQLANQN---FPILITSCLKFIYVYRNQFLKEMLLD
SteCoe_g28374     431 PRNGASSVATSQAHILELYNNLVCPQLANHS---FPILITSCLKFIYVYRNQFLKEMLLD
SacCer_CSE1       439 TNAGVSS-TNNLLNVVDFFTKEIAPDLTSNNIPHI-ILRVDAIKYIYTFRNQLTKAQLIE
DroMel_CSE1       447 QKHGITQ-TSELVPLPEFCAQQIIPELERPNINEFPVLKAAAIKYVMVFRSILGPQVLAS
HomSap_CAS        444 QKHGITQ-ANELVNLTEFFVNHILPDLKSANVNEFPVLKADGIKYIMIFRNQVPKEHLLV
MusMus_XPO2       444 QKHGITQ-ANELVNLTEFFVNHILPDLKSNNVNEFPVLKADGIKYIMIFRNQVPKEHLLV

SteCoe_g28919+8   516 IMGKVIGFLDSNNVLLASYAAATLERLLMIKNDKD---LIFTKDFLSSCLNQLLQNIAMA
SteCoe_g28374     488 IMGKVIGFLDSNNVLLASYAAATLERLLMIKNDRD---LIFTKEFLSSCLNQLLQSIAMA
SacCer_CSE1       497 LMPILATFLQTDEYVVYTYAAITIEKILTIRESNTSPAFIFHKEDISNSTEILLKNLIAL
DroMel_CSE1       506 CLPQLIRHLPAESSVVHSYAACSVEKILSMRDASN--AIVFGPQILAPYTTELISGLFAT
HomSap_CAS        503 SIPLLINHLQAESIVVHTYAAHALERLFTMRGPNN--ATLFTAAEIAPFVEILLTNLFKA
MusMus_XPO2       503 SIPLLISHLEAESIVVHTYAAHALERLFTMRGSNN--TTLFTAAEIAPFVEILLTNLFKA

SteCoe_g28919+8   573 LQKHP-------KNTYIMNAFFRVIWISQDLFSSFAVPACDIFINYIKQVLSEPQSSEPH
SteCoe_g28374     545 LQKHP-------KNTYIMNAFFRVIWISQDLFSSFAVPACDIFISYIKQVLSEPQSSEPH
SacCer_CSE1       557 ILKHGSSPEKLAENEFLMRSIFRVLQTSEDSIQPLFPQLLAQFIEIVTIMAKNP--SNPR
DroMel_CSE1       564 LSLPGSG-----ENEYVMKAIMRSFSVLQSAAMPFMGVALPRLTEILTQVAKNP--SRPQ
HomSap_CAS        561 LTLPGSS-----ENEYIMKAIMRSFSLLQEAIIPYIPTLITQLTQKLLAVSKNP--SKPH
MusMus_XPO2       561 LTLPGSS-----ENEYIMKAIMRSFSLLQEAIIPYIPTLITQLTQKLLAVSKNP--SKPH

SteCoe_g28919+8   626 FNWLLFECIALAMKWSG----PGISDIQKKIEPYMALIIQKSNADLLPYAFQIQAFFIRL
SteCoe_g28374     598 FNWLLFECIALAMKWSG----PGISDIQKKLEPYMALIIQKSNADLLPYAFQIQAFFIRL
SacCer_CSE1       615 FTHYTFESIGAILNYTQRQNLPLLVD---SMMPTFLTVFSEDIQEFIPYVFQIIAFVVEQ
DroMel_CSE1       617 FNHYLFETLALCIKIVCHADSSAVSSFEEALFPVFQGILQQDIVEFMPYVFQMLSVLLEM
HomSap_CAS        614 FNHYMFEAICLSIRITCKANPAAVVNFEEALFLVFTEILQNDVQEFIPYVFQVMSLLLET
MusMus_XPO2       614 FNHYMFEAICLSIRITCKANPAAVVNFEEALFLVFTEILQNDVQEFIPYVFQVMSLLLET

SteCoe_g28919+8   682 L---INLSQTNQNLISSILPIDNWESGSRYYLPTLVIFLENLLATNASSM----APQISA
SteCoe_g28374     654 L---SSISQTNQNLISSILPIDNWESGSRYYLPTLVIFLENLLMTNASSM----APQISA
SacCer_CSE1       672 S---ATIPESIKPLAQPLLAPNVWELKGN--IPAVTRLLKSFIKTDSSIF-----PDLVP
DroMel_CSE1       677 REGTGTIPEPYWALFPCLLSPALWDRTGN--VTPLIRLISAFIKQGSAQI--QALGKLSG
HomSap_CAS        674 HK--NDIPSSYMALFPHLLQPVLWERTGN--IPALVRLLQAFLERGSNTIASAAADKIPG
MusMus_XPO2       674 HK--NDIPSSYMALFPHLLQPVLWERTGN--IPALVRLLQAFLERGSSTIATAAADKIPG

SteCoe_g28919+8   735 LCNIAHKLF-NLGLDGQAFSLLTTLIETYAFENLHPYMHPIYLIIFTKLHNSKSQNIRLS
SteCoe_g28374     707 LCNIAHKLF-TLGLDGQAFSLLTTLIETYSFENLHPYMHPIYMIIFTKLHNSKSQNIRLS
SacCer_CSE1       722 VLGIFQRLIASKAYEVHGFDLLEHIMLLIDMNRLRPYIKQIAVLLLQRLQNSK------T
DroMel_CSE1       733 ILGIFQKMIASKANDHEGFYLLQNLLSYYPPAEIQTNLRQIFGLLFQRLSLSK------T
HomSap_CAS        730 LLGVFQKLIASKANDHQGFYLLNSIIEHMPPESVDQYRKQIFILLFQRLQNSK------T
MusMus_XPO2       730 LLGVFQKLIASKANDHQGFYLLNSIIEHMPPESVDQYRKQIFILLFQRLQNSK------T

SteCoe_g28919+8   794 PRFHKGSILFVSSVILKYGWKVLSDSMNSVQPGIFFMLVKGQILQNLRSIETIVERRAVI
SteCoe_g28374     766 PRFHRGSIMFVSSFILKYGWNVLSDSMNSVQPGIFFMLVKGQILQNLRSIETIVERRAVI
SacCer_CSE1       776 ERYVKKLTVFFGLISNKLGSDFLIHFIDEVQDGLFQQIWGNFIITTLPTIGNLLDRKIAL
DroMel_CSE1       787 PKYLSGIIIFFSFYVIKFSGSQMAQLIDEIQPNLFGMLLDRVFITEMGKIPKEQDRKMVA
HomSap_CAS        784 TKFIKSFLVFINLYCIKYGALALQEIFDGIQPKMFGMVLEKIIIPEIQKVSGNVEKKICA
MusMus_XPO2       784 TKFIKSFLVFINLYCIKYGALALQEIFDGIQPKMFGMVLEKIIIPEIQKVSGNVEKKICA
```

```
SteCoe_g28919+8  854 LAMSVLMQNI-----EVTNELWINIVIS-VSKILDSTTNILSGTVYSNGLVDLPEENTIQ
SteCoe_g28374    826 LAMSALMQNT-----EVTNELWINIVIS-VSKILDSTTNILSGTVYSNGLVDLPEENTIQ
SacCer_CSE1      836 IGVLNMVING-----QFFQSKYPTLISSTMNSIIETASSQSIA-NLKNDYVDLDNLEEIS
DroMel_CSE1      847 VGVTKLLTETPEILQQQYATFWPRLLHS-LIDLFERPPEKLMG--LEIGETAGVAEDPDA
HomSap_CAS       844 VGITKLLTECPPMMDTEYTKLWTPLLQS-LIGLFELPEDDTIP-----DEEHFIDIEDTP
MusMus_XPO2      844 VGITKLLTECPPMMDTEYTKLWTPLLQS-LIGLFELPEDDSIP-----DEEHFIDIEDTP

SteCoe_g28919+8  908 MTRDSFQKVYSTEMPLTDKYASL---PNEKMFFINTICS-QQYPTGSFWSFAGQHLDQQV
SteCoe_g28374    880 MTRDSFQKVYSAEIPLTDKYPHL---PNEKMFFINTICN-QQYPTGSFWSFAGQHLDQQI
SacCer_CSE1      890 TFGSHFSKLVSISEKPFDPLPEIDVNNGVRLYVAEALNKYNAISGNTFLNTILPQLTQEN
DroMel_CSE1      904 GYQVAFAQLTHAQPNQQDHLAEI---KDARQFLATSLSKFAQARAGEFSTLLSP-LEPEY
HomSap_CAS       898 GYQTAFSQLAFAGKKEHDPVGQM--VNNPKIHLAQSLHKLSTACPGRVPSMVSTSLNAEA
MusMus_XPO2      898 GYQTAFSQLAFAGKKEHDPVGQM--VNNPKIHLAQSLHKLSTACPGRVPSMVSTSLNAEA

SteCoe_g28919+8  964 PTILNRYAQLFNQQIR
SteCoe_g28374    936 PTILNRYAQLFNQQIR
SacCer_CSE1      950 QVKLNQLLVGN-----
DroMel_CSE1      960 KQVLQKYCDQAGVRIA
HomSap_CAS       956 LQYLQGYLQAASVTLL
MusMus_XPO2      956 LQYLQGYLQAASVTLL
```

Data S2: Multiple alignment of CSE1 genes, related to Figure 2.

Multiple alignment of both *Stentor coeruleus* CSE1 orthologs and CSE1 homologs from *S. cerevisiae, D. melanogaster, H. sapiens, and M. musculus.* Multiple alignment performed in MEGAX using MUSCLE, displayed using Boxshade. The N-terminal half of CSE1 has multiple contact sites with importin alpha and is generally more conserved.[48,49] In *S. cerevisiae* CSE1, the region from Asn346 to Asn379 is necessary for forming a complex with Ran-GTP and importin-alpha, and acts as a hinge between the N and C terminal halves of CSE1 (Green box).[49] The tryptophan at position 419 in S. cerevisiae CSE1 is conserved in other CSE1 homologs as well as related proteins like importin betas.[49] This tryptophan is also conserved in *Stentor* CSE1 (magenta box). Asp220 in *S. cerevisiae* CSE1 has been shown to be necessary for CSE1 to form a complex with importin alpha and RanGTP – this residue is also conserved in *Stentor* CSE1 (blue box).[50]

Supplemental Movie S1: Macronucleus of a rotating *Stentor*, related to Figure 1.
Movie of a freely swimming and rotating stentor with its macronucleus stained with Hoechst 33342. The video is played back at 0.5x speed to make it easier to visualize the macronuclear nodes.
